# Novel Protein Mg2046 Regulates Magnetosome Synthesis in *Magnetospirillum gryphiswaldense* MSR-1 by Modulating a Proper Redox Status

**DOI:** 10.3389/fmicb.2019.01478

**Published:** 2019-06-26

**Authors:** Xu Wang, Haolan Zheng, Qing Wang, Wei Jiang, Ying Wen, Jiesheng Tian, Jianbo Sun, Ying Li, Jilun Li

**Affiliations:** ^1^State Key Laboratory of Agrobiotechnology, College of Biological Sciences, China Agricultural University, Beijing, China; ^2^Guangdong Provincial Key Laboratory of Stomatology, Guanghua School of Stomatology, Hospital of Stomatology, Sun Yat-sen University, Guangzhou, China

**Keywords:** *Magnetospirillum gryphiswaldense*, Mg2046, magnetosome, regulator, redox status, dissimilatory denitrification pathway

## Abstract

Magnetotactic bacteria (MTB) are a large, polyphyletic group of aquatic microorganisms capable of absorbing large amounts of iron and synthesizing intercellular nano-scaled nanoparticles termed magnetosomes. In our previous transcriptomic studies, we discovered that a novel gene (*MGMSRv2_2046*, termed as *mg2046*) in *Magnetospirillum gryphiswaldense* strain MSR-1 was significantly up-regulated during the period of magnetosome synthesis. In the present study, we constructed a MSR-1 mutant strain with deletion of *mg2046* (termed *Δmg2046*) in order to evaluate the role of this gene in cell physiological status and magnetosome formation process. In comparison with wild-type MSR-1, *Δmg2046* showed similar cell growth, but much lower cell magnetic response, smaller number and size of magnetosomes, and reduced iron absorption ability. *mg2046* deletion evidently disrupted iron uptake, and redox equilibrium, and strongly inhibited transcription of dissimilatory denitrification pathway genes. Our experimental findings, taken together with results of gene homology analysis, indicate that Mg2046 acts as a positive regulator in MSR-1 under microaerobic conditions, responding to hypoxia signals and participating in regulation of oxygen metabolism, in part as a co-regulator of dissimilatory denitrification pathway with oxygen sensor MgFnr (MGMSRv2_2946, termed as Mg2946). Mg2046 is clearly involved in coupled regulation of cellular oxygen, iron and nitrogen metabolism under micro-aerobic or anaerobic conditions. Our findings help explain how MSR-1 cells initiate dissimilatory denitrification pathway and overcome energy deficiency under microaerobic conditions, and have broader implications regarding bacterial survival and energy metabolism strategies under hypoxia.

## Introduction

Magnetotactic bacteria (MTB) are a polyphyletic group of prokaryotes found in aquatic and sedimentary environments worldwide (Faivre and Schüler, 2008). A unique characteristic of MTB is the ability to synthesize magnetosomes [nano-sized and single magnetic domain crystals of magnetite (Fe_3_O_4_) or greigite (Fe_3_S_4_), arranged in chains and enveloped by a lipid bilayer membrane] under oxygen-limited conditions (Lefevre and Bazylinski, 2013). Magnetite formation by MTB is up to 10^8^ kg/year (Lin et al., 2014). MTB play a key role in environmental iron cycling in view of their worldwide distribution and capacity for iron assimilation into magnetosome. They provide a useful model for studies of microbial orientation and navigation, and a source of natural nanomaterials.

Mechanisms of magnetosome formation have been studied for many decades. Clustering of *mam* and *mms* (magnetosome membrane protein genes) typically observed in magnetosome island (MAI, part of MTB genome), is genetic determinant of magnetite biomineralization (Komeili, 2012). This sophisticated, stepwise process has been extensively studied based on deletion of single, multi, or full operon of these genes (Murat et al., 2010; Quinlan et al., 2011; Raschdorf et al., 2013; Lohse et al., 2014, 2016). Functions of Mam proteins (e.g., MamA, MamP) in Mam complex assembly and iron mineralization have been examined *in vitro* using various approaches (Zeytuni et al., 2011; Jones et al., 2015). There has been increasing interest in the roles of genes involved in iron absorption and regulation of magnetosome formation. Genes of *feoAB1* operon control transport of ferrous iron (Fe^2+^) and play an accessory role in magnetosome formation (Rong et al., 2008; Kolinko et al., 2014). Our previous studies demonstrated that the transcriptional regulator protein Fur (Ferric uptake regulator) regulates iron metabolism genes (including *feoAB*) and affects magnetosome formation in the well-known MTB, *Magnetospirillum gryphiswaldense* MSR-1 (Qi et al., 2012; Deng et al., 2015). Schüler’s group showed that periplasmic nitrate reductase, terminal oxidase *cbb3*, and oxygen sensor MgFnr (fumarate and nitrate reduction regulator in *M. gryphiswaldense*) are also involved in the biomineralization process (Li et al., 2012, 2013, 2014a). We reported recently that a series of redox enzymes and sensors, including OxyR-like (an H_2_O_2_ sensor-like protein) play key roles in magnetosome formation (Zhang et al., 2017). Steadily increasing experimental evidence shows clearly that magnetosome synthesis in MTB is controlled by not only *mam/mms* genes but also various genes related to basic cellular metabolism in MTB.

We applied transcriptome analysis to explore relationships between magnetosomes and cell physiological status (Wang et al., 2016, 2017). Transcription levels of genes involved in certain metabolic pathways, e.g., dissimilatory denitrification, terminal oxidase, and ferrous uptake genes, were affected by changes in dissolved oxygen levels (Wang et al., 2016). Other studies have shown that these pathways are involved in magnetosome formation (Rong et al., 2008; Li et al., 2012, 2014a). Several unknown proteins and their genes showed notably increased or reduced expression under hypoxic (oxygen-deficient) or iron-rich conditions. Among these, gene MGMSRv2_2046 (here termed *mg2046*) showed significant 5.6-fold upregulation (in association with magnetosome formation), and RPKM (reads per kilobase of transcript, per million mapped reads) values 45.23 and 8.14 (*p*-value 0.048 < 0.05) under high- and low oxygen conditions, respectively.

In this study, we constructed a MSR-1 *mg2046* deletion mutant (termed *Δmg2046*), and compared wild-type (WT) vs. mutant strains in terms of magnetosome synthesis and cellular physiology. Our findings suggest that Mg2046 is involved indirectly in the early stage of magnetosome synthesis, and directly in the mature stage through regulation of various metabolic pathways – particularly redox reactions driven by terminal oxidases and dissimilatory denitrification. Such pathways involve appropriate redox state, and affect cellular iron absorption and energy production required for magnetosome formation. Mg2046 is an oxygen-sensitive regulator: its synthesis and activity are inhibited by aerobic conditions. Microaerobic conditions are necessary for Mg2046 activity, which indirectly regulates the MSR-1 biomineralization process.

## Materials and Methods

### Strains and Culture Conditions

Bacterial strains and plasmids used in this study are listed in Table 1.

**Table 1:** Strains and plasmids used in this study.

Strains and Plasmids	Characters	Source
*Strains*		
MSR-1 WT	Wild-type *Magnetospirillum gryphiswaldense*: Nx^r^	DSM6361
MSR-1 *Δmg2046*	MGMSRv2_2046 deficient mutant: Nx^r^, Gm^r^	This study
MSR-1 *Com_mg2046*	MGMSRv2_2046 gene complementary strains of *Δmg2046* mutant: Nx^r^, Gm^r^, Tc^r^	This study
*Escherichia coli* DH5α	*endA1 hsdR17* [r-m+] *supE44 thi-1 recA1 gyrA* [NalR] *relA relA1* Δ[*lacZYA-argF*] *U169 deoR* [∅80Δ (*LacZ*) M15]	Novagen
*E. coli* DH5α *Com_mg2046*	*E. coli* DH5α containing pMD18-T-MGMSRv2_2046	This study
*E. coli* S17-1	*Thi endA recA hsdR* with RP4-2-Tc::Mu-Km::Tn7 integrated in chromosome; Sm^r^, Tra^+^	Novagen
*E. coli* S17-1 *Com_mg2046*	*E. coli* S17-1 containing pRK415-MGMSRv2_2046; Nx^r^, Tc^r^	This study
*Plasmids*		
pMD18-T	Cloning vector; Amp^r^	Takara
pUCGm	Containing Gm cassette, Gmr, Amp^r^	Our lab
pUX19	Suicide vector for *M. gryphiswaldense* MSR-1, Kan^r^	Our lab
pUXsuc_mg2046	pUX19 containing MSR-1 MGMSRv2_2046 upstream and downstream region, Gm cassette, Kan^r^, Gm^r^	This study
pBBR1MCS-2^a^	Broad-host range lacZ promoter vector; Km^r^	Our lab
pBBR1MCS-2_mg2046	pBBR1MCS-2 containing MGMSRv2_2046, Km^r^	This study

*^*a*^Kovach et al. (1995).*

*Magnetospirillum gryphiswaldense* MSR-1 (DSM No. 6361) was originally purchased from Deutsche Sammlung von Mikroorganismen und Zellkulturen (Brunswick, Germany) and has been cultivated subsequently for >10 years in our lab. MSR-1 cells were cultured in sodium lactate medium (SLM) at 30°C with shaking (100 rpm). SLM contained (per L) 2.60 g sodium lactate solution (55–65%), 1.02 g NaNO_3_, 0.50 g K_2_HPO_4_⋅3H_2_O, 0.10 g MgSO_4_, 0.10 g yeast extract, and 5.00 mL trace element mixture (Rong et al., 2008). Sterilized ferric citrate was added (final concentration 60 μM) after autoclaving. For conjugation experiments, MSR-1 were cultured in solid selecting medium, which contained 4.00 g/L sodium glutamate as N source instead of NaNO_3_ and yeast extract, and 15 g/L agar. MSR-1 strains were cultured in 100-mL serum bottles filled with 50 mL medium. *E. coli* strain was cultured in Luria broth (LB) at 37°C with shaking (200 rpm). Antibiotics were used at the following concentrations (μg/mL): (for *E. coli*) ampicillin (Amp) 100, kanamycin (Km) 50, chloramphenicol (Cm) 50, gentamicin (Gm) 20; (for MSR-1) nalidixic acid (Nx) 5, Km 5, Cm 5, Gm 5.

### Construction of *mg2046* Mutant and Its Complementary Strain

**FIGURE 1:**
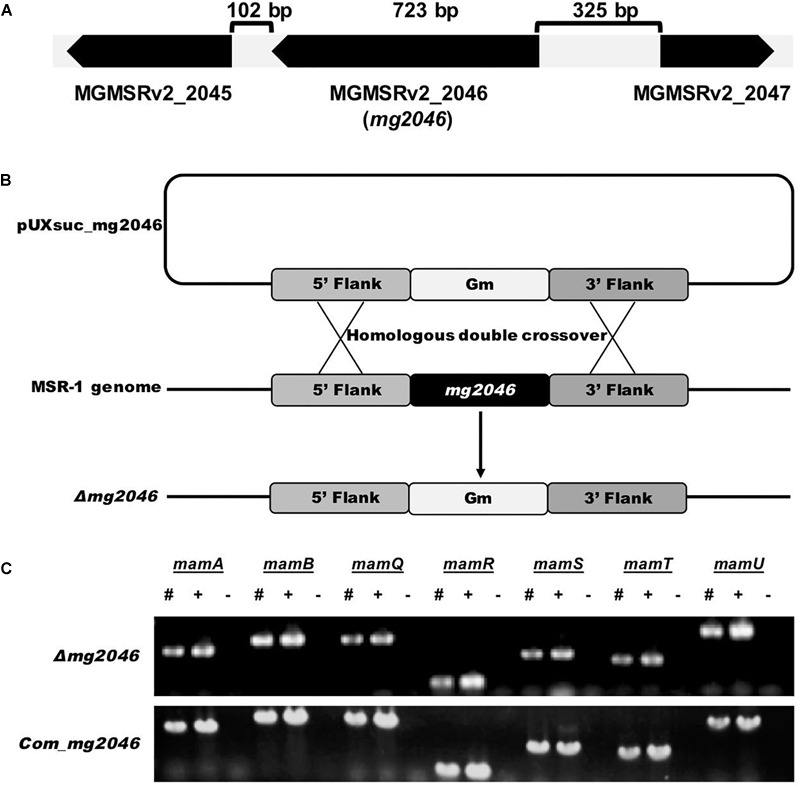
Construction and verification of *mg2046* null strain. **(A)** Schematic representation of *mg2046* and flanking genes in MSR-1 genome. Arrows: genes. Brackets: interval gaps between genes. **(B)** Schematic representation of homologous double-crossover. pUXsuc_mg2046: suicide plasmid. Following double-crossover, *mg2046* gene was replaced by gentamicin gene in MSR-1 genome. **(C)** Detection of *mam* genes in mutant and complementary strain by PCR. #, Mutant genome; +, complementary strain genome; –, ddH_2_O used as template. Mutant and complementary strains were successfully constructed without auto-deletion of *mam/mms* genes.

Related genes loci in the genome are illustrated in Figure 1A. Recombinant MSR-1 strain was constructed by biparental conjugation as described previously (Rong et al., 2008). For construction of *mg2046*-deficient strain, upstream and downstream fragments were amplified and ligated, along with gentamicin resistance cassette from digested pUCGm, into suicide vector pUX19 to form suicide plasmid pUXsuc_mg2046. This plasmid was transformed into MSR-1 by conjugation using *E. coli* S17-1 as donor strain (Figure 1B). Colonies that showed growth in Gm^r^/Nx^r^ selecting medium but not in Gm^r^/Nx^r^/Km^r^ selecting medium were considered putative double-crossover strains. Clones were confirmed by PCR. Clones in which *mam*/*mms* could be amplified, but *mg2046* could not were selected as *mg2046* mutants. For construction of complementary strain, *mg2046* containing enzyme site amplified by primer were cut and ligated into pBBR1MCS-2 plasmid (Kovach et al., 1995). The recombinant plasmid was transformed into *mg2046* mutant by biparental conjugation as above. Clones showing growth in Gm^r^/Nx^r^/Km^r^ selecting medium were confirmed by PCR with *mg2046* and *mam/mms* gene primers (Figure 1C).

### Cell Growth, Magnetic Response, and TEM Observation

Cell growth and magnetic response were estimated, respectively, as OD_565_ of culture broth and Cmag value, as described previously (Zhao et al., 2007; Rong et al., 2008). Cells were cultured in SLM for 24 h, washed twice with ddH_2_O, suspended to a concentration with OD_565_ ∼1, and dropped onto copper grids. Samples were air-dried and observed directly by TEM (model JEM-1230, JEOL; Tokyo, Japan). Numbers and diameters of magnetosomes were analyzed statistically using ImageJ software program (National Institutes of Health; Bethesda, MD, United States). Lattice structure of magnetosomes was observed by high-resolution TEM (model JEM-2100, JEOL).

### Iron Absorption Ability and Intracellular Iron Content

Cells were grown in SLM for 24 h at 30°C, and harvested by centrifugation at 4000×*g* and 4°C for 20 min. The pellet was washed with equivalent volume of PBS and dried to constant weight. Cells were digested by nitric acid, and total cellular iron content was measured by inductively coupled plasma-optical emission spectrometry (ICP-OES) (Optima 5300 DV system, PerkinElmer; Waltham, MA, United States) (Rong et al., 2008). Residual iron concentration was measured by ferrozine method in broth supernatant aspirated after centrifugation (Dailey and Lascelles, 1977).

### Magnetic Properties

Cells were cultured and harvested as in the above section, and room-temperature hysteresis loops and first-order reversal curves (FORCs) were measured using a VSM3900 magnetometer (Princeton Measurements Corp.; Westerville, OH, United States; sensitivity 5 × 10^-10^ Am^2^). Saturation magnetization, saturation remanence, and coercivity were determined as described previously (Li et al., 2010), with slight modification of methods.

### Real-Time Quantitative PCR (qPCR)

Cells were cultured and harvested as in the preceding sections. Total RNA was isolated using TRIzol reagent (Tiangen Biotech; Beijing, China), and genome DNA in total RNA was digested by DNase I (Takara; Shiga, Japan). DNA remaining in total RNA was detected by PCR using digested product as template. RNA was then reverse-transcribed into cDNA using Moloney murine leukemia virus (M-MLV) reverse transcriptase (Promega; Madison, WI, United States). Transcriptional abundance of genes in samples was quantified by qPCR, using housekeeping gene rpoC (encodes RNA polymerase subunit β′) as internal control. qPCR was performed using LightCycler 480 RT-PCR system and LightCycler 480 SYBR green I Master kit (Roche; Mannheim, Germany) as per manufacturer’s instructions. Selected genes and primer sequences are listed in Supplementary Table S1. Relative transcription levels of genes were determined by 2^-ΔΔCp^ method (Zhang et al., 2012; Wang et al., 2015).

### Dissimilatory Nitrate Reductase Detection

Cells were cultured and harvested as in the preceding sections. Dissimilatory nitrate reductase enzyme activity of tested bacterial cells was analyzed using bacterial dissimilatory nitrate reductase activity colorimetric assay kit GMS15031.1.3 v.A (Genmed Scientifics; Wilmington, DE, United States) as per manufacturer’s instructions.

### Bioinformatic Analysis

Selected MSR-1 genes were identified by reference to complete genome sequences (*M. gryphiswaldense* MSR-1 v2) found at MicroScope website^1^ (note: gene IDs mentioned in this report were taken from this website) or NCBI (GenBank Accession: NC_023065) (Wang et al., 2014).

## Results and Discussion

### Mg2046 Is a Novel Regulatory Protein and Is Conserved in Genus *Magnetospirillum*

Gene *MGMSRv2_2046* (*mg2046*) is located from 2160600 to 2161322 nt (723 bp) in the complete *M. gryphiswaldense* MSR-1 genome (MGMSRv2), and was annotated as putative transcriptional activator FnrA (fumarate and nitrate reduction regulator A). Upstream gene *MGMSRv2_2047, mg2046*, and downstream gene *MGMSRv2_2045* are located at intervals of 325 and 102 bp without overlap (Figure 1A). Transcriptional orientation of each gene is indicated by arrows. *mg2046* encodes a protein of 240 amino acids.

Fnr-family proteins (whose members include FnrA and FnrL) belong to the Crp/Fnr superfamily, which contributes to the metabolic versatility of bacteria (Körner et al., 2003). Fnr and Crp (cAMP receptor protein) are the most common members of this superfamily. Searches of genomes of other bacteria showed that Mg2046 has low identity of 29.44% with Fnr from *Escherichia coli* K12, 29.38% with FnrA from *Pseudomonas stutzeri*, 28.64% with Fnr from *Vibrio cholera*, and had similarities to items in the MaGe annotation platform of SwissProt (Table 2). According to NCBI’s Blastp program on NCBI, MGMSRv2_2046 is predicted to have specific hits of cd00038: CRP_ED domain in N-terminus and pfam13545: HTH_Crp_2 domain in C-terminus, suggesting that it is a Crp. However, Mg2046 has low identity (32.43%) with Crp from *E. coli* K12 (Table 2). Mg2046 can therefore not be identified simply as a Crp or Fnr protein. On the other hand, Mg2046 homologs have identities with proteins from other *Magnetospirillum* species of 66.95% with MAGMOB_540028 from *M. moscoviense* BB-1, 60.09% with amb2977 from *M. magneticum* AMB-1, 59.21% with JXSL01_v1_280147 from *M. magnetotacticum* MS-1, 58.08% with MAGMAS_370140 from *M. marisnigri* SP-1, and 57.89% with AONQv1_290020 from *Magnetospirillum* sp. SO-1 (Table 2). These findings indicate that Mg2046 homologs are highly conserved in genus *Magnetospirillum*.

**Table 2:** Crp/Fnr proteins, Mg2046 homologs, and UniProt IDs.

Protein	Strain	UniProt ID	Identity (%)	Length (aa)
Fnr	*Escherichia coli* K12	P0A9E5	29.44	250
FnrA	*Pseudomonas stutzeri*	P47200	29.38	244
Fnr	*Vibrio cholerae* serotype	A5F890	28.64	250
Crp	*E. coli* K12	P0ACJ8	32.43	209
Mg2046 homolog	*Magnetospirillum gryphiswaldense* MSR-1 v2	V6F4P2	100	240
	*M. moscoviense* BB-1	A0A178N0G1	66.95	243
	*M. magneticum* AMB-1	Q2W2Z4	60.09	246
	*M. magnetotacticum* MS-1	A0A0C2YTE7	59.21	240
	*M. marisnigri* SP-1	A0A178MVM6	58.08	248
	*Magnetospirillum* sp. SO-1	M2ZQS4	57.89	246

*Identity values were calculated based on alignment of proteins with Mg2046.*

### Construction of Mutant Strain *Δmg2046*

To investigate the role of Mg2046 in MSR-1 cell growth and magnetite biomineralization, we constructed null mutant strain *Δmg2046* using homologous double-crossover strategy as described previously (Wang et al., 2015; Figure 1B). *mam*/*mms* genes were detected by PCR, in consideration of their possible auto-deletion during double-crossover of the target gene (Figure 1C). Upstream and downstream regions (up to 1 kb) of *mg2046* in the mutant were sequenced, confirming that there were no other changes. The genes flanking *mg2046* were also investigated. The *mg2045* transcribes in the same direction with *mg2046*, while the *mg2047* transcribes in the opposite direction to *mg2046*. The relative transcription levels of *mg2045* in the mutant and the wild type strain were analyzed by qPCR, and no significant difference was found.

A complementary strain was constructed, *mam/mms* genes were again confirmed by PCR, and sequencing of adjacent regions demonstrated that there were no other changes (Figure 1C). We did not observe recovery of magnetic response in the mutant back to WT level. That is, transfer of *mg2046* complementary plasmid did not recover the magnetosome formation ability back to WT phenotype. qPCR analysis showed that *mg2046* was overtranscribed >60-fold in *Δmg2046* relative to WT (Supplementary Figure S1). Expression of complementary gene was too high in the receptor. One possible explanation is that gene copies, promoters, and regulatory processes were distinctive in plasmid vs. genome structure, resulting in different transcription and expression levels. Overexpression of *mg2046* disrupted the metabolic balance of cells. On the other hand, magnetosome formation requires proper expression of Mg2046, triggered by low oxygen conditions. Schüler’s group reported a similar phenomenon when MgFnr (regulator of dissimilatory denitrification) was overexpressed (Li et al., 2014b). Therefore, the complementary strain was not used for subsequent experiments.

### *Δmg2046* Cells Grow Normally but Display Low Magnetic Response

**FIGURE 2:**
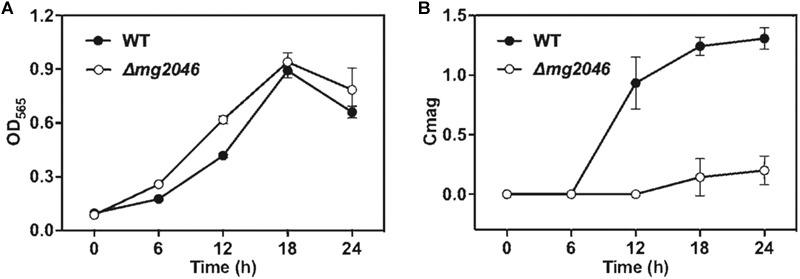
Cell growth and magnetic response of WT and *Δmg2046*. **(A)** Cell growth (OD_565_). **(B)** Magnetic response (Cmag). Growth was comparable for the two strains, but magnetic response was much lower for *Δmg2046*.

WT and *Δmg2046* were cultured under microaerobic conditions, with medium and culture conditions similar to those in our previous studies. As Mg2046 belongs to the Fnr family, and Fnr is one of the major regulatetors of dissimilatory denitrification pathway, ammonium in the medium was replaced by sodium nitrate at equal concentration. Samples were taken every 6 h, and cell growth and magnetic response were estimated, respectively, by OD_565_ and Cmag. Cell growth curves of *Δmg2046* and WT were similar, with maximal OD_565_ (at 18 h) 0.94 ± 0.05 and 0.89 ± 0.04, respectively (Figure 2A), indicating that *mg2046* deletion had no notable effect on cell growth. In contrast, Cmag curves for the two strains were very different. Cmag for WT was 0.93 ± 0.22 at 12 h and 1.31 ± 0.09 at 24 h, whereas Cmag for *Δmg2046* was 0.14 ± 0.16 at 18 h and only 0.20 ± 0.12 at 24 h (Figure 2B). The reasons for the strikingly lower Cmag values of *Δmg2046* were explored in subsequent experiments, as described in the following sections.

### Synthesis of Abnormal Magnetosome Chains in *Δmg2046*

**FIGURE 3:**
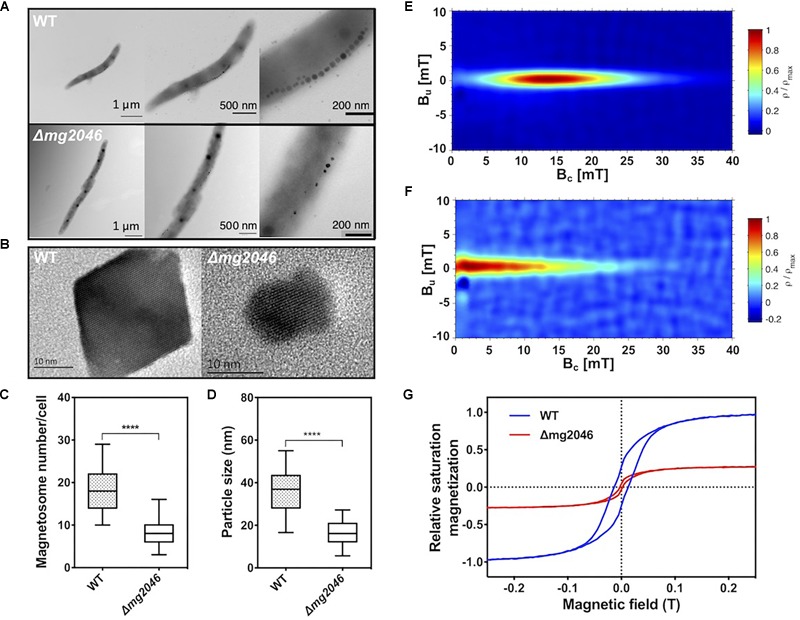
Characteristics of magnetosomes synthesized by WT and *Δmg2046*. **(A)** TEM images, with progressive magnification from left to right. Bars: 1 μm, 500 nm, 200 nm. **(B)** High-resolution TEM image of nanoparticles. Both strains showed typical ferroferric oxide lattice structure. Bar: 10 nm. **(C,D)** Box-plot charts showing statistical analysis of magnetosome diameter and number. ^∗∗∗∗^*p* < 0.05. **(E)** FORCs diagram of WT. **(F)** FORCs diagram of *Δmg2046*. **(G)** Relative saturation magnetization based on comparison of hysteresis loops of WT (blue) and *Δmg2046* (red). Max movement of WT is defined as ±1. Magnetosomes synthesized by *Δmg2046* were fewer, smaller, and more weakly magnetic.

Cultured WT and *Δmg2046* cells were collected at 24 h and observed by transmission electron microscopy (TEM). Magnetosomes in WT had normal appearance, and formed a regular linear chain along the long axis of the cell, whereas those in *Δmg2046* were fewer in number, irregularly shaped, and had large spaces between particles (Figure 3A). High-resolution TEM revealed that *Δmg2046* had smaller particles but had typical ferroferric oxide lattice structure similar to that of WT (Figure 3B). Average magnetosome number per cell (8.30 ± 3.02 vs. 18.11 ± 4.89) and magnetosome particle size (16.50 ± 5.26 vs. 36.10 ± 9.17 nm) were much smaller for *Δmg2046* than for WT (details in Supplementary Table S2). In box-plot charts, minimum (low bar end), majority (box), and maximum (high bar end) values for these two parameters were also significantly smaller (*p* < 0.01) for *Δmg2046* than for WT (*t*-test, *p* < 0.01) (Figure 3C,D).

To elucidate magnetic properties of magnetosome, we measured room temperature first-order reversal curves (FORCs) and hysteresis loops. FORCs diagram for WT showed closed contours with narrow vertical distribution around coercivity value ∼15 mT (Figure 3E), indicating predominant formation of typical single-domain magnetosomes, whereas FORCs diagram for *Δmg2046* showed much lower coercivity value (<5 mT) (Figure 3F). Relative saturation magnetization was compared based on hysteresis loops. Hysteresis loop for WT (Figure 3G; blue line) had “pot-bellied” shape, while that for *Δmg2046* (red line) had “thin-waisted” shape.

Comparison of relative saturation magnetization as above indicates that degree of magnetism is less for *Δmg2046* than for WT. The difference between the two strains appears to be mainly in their magnetic properties. The above findings, taken together, indicate that *mg2046* deletion alters magnetosome synthesis and maturation, resulting in particles that are fewer, smaller, and less magnetic.

### Iron Absorption Rate and Intracellular Iron Content

**FIGURE 4:**
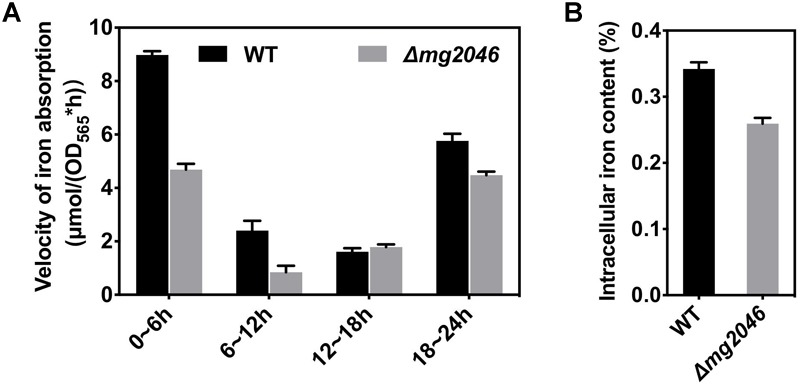
Iron utilization by WT and *Δmg2046*. **(A)** Iron absorption rate. **(B)** Intracellular iron content. Both parameters were lower for *Δmg2046* than for WT.

Reduced iron absorption also can reduce magnetosome synthesis in cells. To estimate iron utilization, WT and *Δmg2046* were cultured under microaerobic conditions with nitrate supplementation, and sampled at 6-h intervals. OD_565_ and iron concentration of culture medium were measured. Iron absorption rate [μmol/(OD_565_^∗^h)] was calculated as [iron concentration (t h)^∗^volume – iron concentration (t h + 6 h)^∗^volume]/{[OD_565_ (t h + 6 h)^∗^volume – OD_565_ (t h)^∗^volume]^∗^6 h}. Iron absorption velocities are compared in Figure 4A. Maximal velocities were observed in the initial 0–6 h period: 8.98 ± 0.14 and 4.86 ± 0.22 μmol/(OD_565_^∗^h) for WT and *Δmg2046*, respectively. Values for both strains declined to ∼2 in the 6–12 h and 12–18 h periods. In the 18–24 h period, values increased to 5.76 ± 0.27 and 4.48 ± 0.13, respectively. Because cells were first grown in trace-ferric medium, and then inoculated into rich-ferric medium, it is possible that they absorbed ferric ion quickly and were ready for magnetosome formation within the initial 6-h period. Comparison of the strains over the entire culture process showed that iron absorption rate was lower for *Δmg2046* than for WT during the 0–6, 6–12, and 18–24 h periods, and similar during the 12–18 h period. Thus, *mg2046* deletion inhibited iron uptake. Intracellular iron contents of the two strains following 24 h were measured. Intracellular iron accounted for 0.34 and 0.26% of cell dry weight in WT and *Δmg2046*, respectively (Figure 4B). Iron content of WT was 1.31-fold higher than that of *Δmg2046*. Absence of *mg2046* results in partial inhibition of iron absorption and reduction of intracellular iron content, such that cells do not have sufficient iron for synthesis of normal magnetosome chains.

The basic requirements for absorption by MTB of iron source rapidly and in large quantity are low-oxygen (microaerobic) and high-iron conditions. The reduction of iron absorption by *mg2046* knockout may conceivably be due to loss of oxygen -regulating ability, insufficient energy, and/or iron supply. To clarify the reasons, we evaluated and compared transcription of selected target genes in WT and *Δmg2046*. The genes and their qPCR primers of selected target genes are listed in Supplementary Table S1.

### Effects of *mg2046* Deletion on Expression of Genes in Various Key Metabolic Pathways

WT and Δmg2046 cultured for 8 and 16 h, and gene transcription were measured by qPCR. Target genes included *mam/mms*, iron transport, terminal oxidase, and dissimilatory denitrification pathway genes.

#### *mam/mms* Genes

**FIGURE 5:**
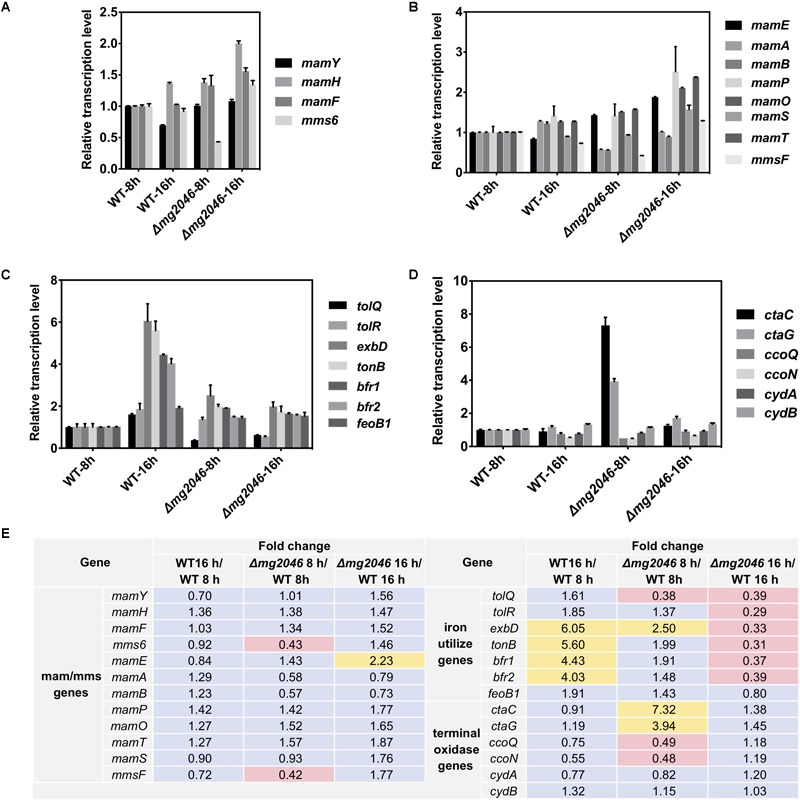
Relative transcription levels of key pathway genes in WT and *Δmg2046*. **(A)** First genes of *mam/mms* operons (see text). **(B)** Important *mam/mms* genes. **(C)** Iron metabolism related genes. **(D)** Terminal oxidase genes. Transcription levels of genes in WT 8 h sample were used as reference (defined as 1) for expression of fold changes in other samples. **(E)** Fold changes between samples. Yellow: fold change >2. Pink: fold change <0.5. *Δmg2046* showed disrupted transcription of *mms*, terminal oxidase, iron uptake, and storage related genes.

Relative transcription levels were measured for *mamY, mamH, mamF, mms6*, and the first genes of *mamXY, mamAB, mamCD*, and *mms* operon (Figure 5A,B). Differences (fold changes in detail) between samples were calculated and summarized in Figure 5E. Values for WT at 8 h were defined as 1. Fold changes of transcription levels ranged from 0.70 to 1.56 (changes <twofold, Figure 5E), except that *mms6* level in *mg2046* was 0.43-fold that in WT at 8 h (Figure 5B). For comparisons of other *mam/mms* genes (*mamE*, -*A*, -*B*, -*P*, -*Q*, -*S*, -*T*, belonging to *mamAB* cluster, and *mmsF* fold changes ranged from 0.58 to 1.87 (changes <twofold, Figure 5E), except for *mamE* fold change 2.23 for *Δmg2046*-16 h vs. WT-16 h, and *mmsF* fold change 0.42 for *Δmg2046*-8 h vs. WT-8 h (Figure 5B,E). Thus, various *mam/mms* genes were stably transcribed at 8 and 16 h, transcription levels differed slightly between WT and *Δmg2046*, and most *mam/mms* genes appeared to be expressed in constitutive form. These findings are consistent with our previous transcriptomic analysis (Wang et al., 2016). *mg2046* deletion had minimal effect on transcription of *mam* genes, but reduced *mms6* and *mmsF* expression in early-stage (0–8 h) magnetosome synthesis, the period when cells absorb large quantities of iron. Functions of *mam*/*mms* genes have been suggested previously to be related to membrane invagination, vesicle formation, ion transport, or control of magnetosome arrangement. Mms6 protein expression is closely related to crystal nucleus formation in magnetosomes. Mms6 was proposed to bind iron ions to control magnetic particle size (Arakaki et al., 2003; Tanaka et al., 2011; Wang et al., 2012). Recent Mms6 structure prediction suggests that its C-terminus is hydrophilic and enriched in acidic amino acid residues, such that Mms6 can aggregate spontaneously on the surface of solution or solid phase to form polymers of varying sizes, which determine magnetic particle size (Arakaki et al., 2016; Staniland and Rawlings, 2016). Other studies suggest that self-assembled MmsF controls magnetite nanoparticle formation *in vitro* (Murat et al., 2011), and is a key regulator of magnetite biomineralization (Rawlings et al., 2014). Our finding that *mg2046* deletion reduces transcription of *mms6* and *mmsF* suggests that Mg2046 protein is involved in regulation of certain pathways and affects the biomineralization process during early stages of cell growth.

#### Iron Metabolism Related Genes

We examined transcription in WT and *Δmg2046*, at 8 and 16 h, of ion transport Tol system genes *tolQ* and *tolR* (Goemaere et al., 2007), iron affinity uptake system TonB-ExbBD component genes *tonB* and *exbB* (Wang et al., 2014; Klebba, 2016), ferrous uptake system gene *feoB1* (Rong et al., 2008), and iron storage protein bacterioferritin *bfr1* and *bfr2* genes (Wang et al., 2016). In WT, transcription levels of these genes were relatively consistent and stable up to 8 h culture. Transcription of *tonB, exbB, bfr1*, and *bfr2* was upregulated >fourfold in WT-16 h compared to WT-8 h. In *Δmg2046*, transcription levels of all genes were unstable during the initial stage (0–8 h), when activity of two iron transport systems is relatively low. All genes except *feoB1* were downregulated (fold change 0.31–0.39) in *Δmg2046*-16 h relative to WT-16 h (Figure 5C,E). Loss of Mg2046 reduced expression of genes related to iron transport. Iron affinity and absorption of *Δmg2046* showed a slight increase at 16h, but this increase was limited and unevenly expressed in comparison with WT.

The TonB-ExbBD system in bacteria is generally a high ferric affinity transport system, and Bfr stores or releases iron to maintain iron balance in cells. Studies of iron utilization and regulation in MSR-1 to date have focused mainly on the ferrous uptake (Feo) system and ferrous uptake regulator (Fur). In the present study, transcription of *tonB, exbD, bfrs*, and *feoB1* were much higher in WT 16 h than in WT 8h. Such increase was not observed in *Δmg2046* 16 h relative to *Δmg2046*-8 h.

In view of the fact that iron utilization and balance in bacteria are co-regulated by Fur family proteins and intracellular ferrous/ferric concentration, the inhibitory effect of TonB, ExbD, and Bfr is presumably exerted in indirect manner, perhaps through disruption of cellular redox balance. MSR-1 has certain iron absorption and regulatory genes (including Tol and TonB-ExbBD system genes) that are ubiquitous in non-magnetic bacteria, but has greater affinity for Fe^3+^ than for Fe^2+^, and does not secrete siderophores (Schüler and Baeuerlein, 1996; Wang et al., 2014); therefore, it may have yet-unknown genes involved in Fe^3+^ absorption (Wang et al., 2016). Coupling of regulation of iron and oxygen metabolism has been clearly demonstrated in MTB. Many aspects of this complex regulatory network remain to be clarified. The present findings indicate that loss of *mg2046* greatly reduces transcription of ferric transport and iron storage related genes during the magnetosome formation process.

#### Terminal Oxidase Operon Genes

The aerobic respiration pathway in MSR-1 has three branches, which allow adaptation and energy production to changing dissolved oxygen levels. When cellular oxygen level decreases to hypoxic conditions, bacterial heme is expressed in large quantities, combines with oxygen with high affinity, and facilitates oxygen transportation and utilization. We examined transcription levels in WT and *Δmg2046*, at 8 and 16 h, of three terminal oxidase operons: *ctaC* and *ctaG* (*ctaCDGE* operon), *ccoQ* and *ccoN* (*ccoNOQP* operon), and *cydA* and *cydB* (*cydAB* operon) (Wang et al., 2016). In WT, transcription of these genes was relatively stable, with slight differences among genes at 16 h, with fold changes in the range 0.55–1.32 (changes <twofold) relative to WT 8 h (Figure 5D,E). In *Δmg2046* 8 h, *ctaC* and *ctaG* were upregulated 7.32–and 3.94-fold, and *ccoQ* and *ccoN* were downregulated 0.48-fold relative to WT 8 h. These findings suggest that in early-stage cell growth intracellular oxygen level fluctuates because of the absence of *mg2046*, and increased amounts of *ctaC* and *ctaG* expression products are required to maintain oxygen balance. In *Δmg2046* 16 h, levels of these genes were more stable, and were only 1.18- to 1.45-fold higher than in WT 16 h (Figure 5D,E). Transcription levels of *cydA* and *cydB* ranged from 0.77 to 1.32 for all comparison sets, indicating more consistent expression in both WT or *Δmg2046* during culture. In summary, *mg2046* deletion had opposite effects on transcription of *cta* and *cco* genes; the former were upregulated and the latter downregulated during early stage (0–8 h) when cells experienced microaerobic culture conditions.

Terminal oxidases are components of the final-step reaction in respiratory chain. Three types of terminal oxidase complex (Cyd, Cta, Cco) are found in *Magnetospirillum*, and Cta and Cco are conserved in strains MSR-1, AMB-1, and SO-1 (Wang et al., 2016). Cco terminal oxidases are involved in maintenance of the proper redox state required for magnetosome formation (Li et al., 2014a). In the present study, *mg2046* deletion resulted in low *cco* expression. Terminal oxidases are regulated by sensing/control system RegBA/PrrBA (the name varies depending on strain) activated by redox state under low-oxygen condition (Bueno et al., 2012). In microarray analysis of *Pseudomonas aeruginosa* genome, RoxSR (component of RegBA/PrrBA) upregulated transcription of *ccoNOQP* operon and downregulated *cox* gene (Cox and Cta are both aa3 terminal oxidases) under low-oxygen conditions (Kawakami et al., 2010; Bueno et al., 2012). *mg2046* deletion thus appears to cause striking changes during early-stage cell growth. Upregulation of *cta* transcription failed to restore normal oxygen balance or iron absorption in cells, and was therefore inadequate to support normal biomineralization process.

Faivre’s group used X-ray absorption spectroscopy and TEM to show that magnetite particles were formed by a reductive process from Fh (Fe_2_O_3_⋅n H_2_O) (Baumgartner et al., 2013). We demonstrated that reducing power (NADH/NAD^+^ ratio) in MSR-1 increased rapidly when a large number of magnetosomes were synthesized (Yang et al., 2013). Redox status clearly plays an important role in magnetosome formation. Dissimilatory denitrification and terminal oxidases (particularly Cco) are closely associated with redox status and reducing power. Kralova et al. (1992) reported that at low redox level dissimilatory denitrification products (dinitrogen or nitrous oxide) were generated, and dinitrogen transformation (the final step of dissimilatory denitrification) was enhanced. In *Rhodobacter sphaeroides*, Cco functions as a redox sensor under aerobic conditions, but enhances reducing power under anaerobic conditions (Oh and Kaplan, 2002). In contrast to bacteria (e.g., *E. coli*) that display only partial dissimilatory denitrification and lack Cta and Cco terminal oxidases, *Magnetospirillum* strains have complete dissimilatory denitrification pathway and multiple types of terminal oxidases (Wang et al., 2016). Thus, redox sensing/regulation in *Magnetospirillum* differs from that in *E. coli*. In *Magnetospirillum*, Mg2046 acts as a redox regulator that promotes *cco* transcription and iron absorption.

#### Dissimilatory Denitrification Pathway Genes

**FIGURE 6:**
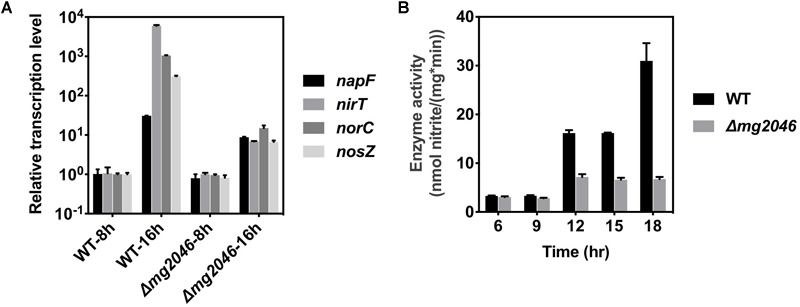
Transcription levels of *nap, nir, nor, nos* genes, and enzyme activity of nitrate reductase, in WT and *Δmg2046*. **(A)** Relative transcription levels of first genes (*nap, nir, nor, nos*) of four operons of dissimilatory denitrification pathway (see text) in WT 8 h, WT 16 h, *Δmg2046* 8 h, and *Δmg2046* 16 h. **(B)** Nitrate reductase activity at times 6, 9, 12, 15, and 18 h.

This pathway has four operons (*nap, nir, nor, nos*; the first genes are, respectively, *napF, nirT, norC, nosZ*) and is responsible for efficient transformation of nitrate → nitrite → nitric oxide → nitrous oxide → nitrogen. Several previous studies indicate that dissimilatory denitrification genes are strongly upregulated under microaerobic conditions and play essential roles in magnetosome formation (Li et al., 2012, 2013; Wang et al., 2016). In the present study, transcription levels of the above four genes in *Δmg2046* vs. WT were similar during the 0–8 h period, but differed significantly during the 8–16 h period (Figure 6A; details in Supplementary Table S3). The levels were 10- to 10^3^-fold higher in WT 16 h than in WT 8 h. In *Δmg2046* 16 h, transcription levels of *napF, nirT, norC*, and *nosZ* were much lower (respectively, 0.28, 1.13 × 10^-3^, 1.42 × 10^-2^, and 2.16 × 10^-2^ fold) than those in WT 16 h. These findings indicate that in middle- and late-stage cell growth (magnetosome maturation stage), the dissimilatory denitrification pathway is strongly activated, and that *mg2046* deletion blocks such activation, particularly in the last three steps. We further investigated activity of nitrate reductase, the key catalytic enzyme in first step. Samples were taken from the two strains at 3-h intervals after 6 h culture. In WT, enzyme activity [nmol nitrate/(mg^∗^min)] was ∼3.0 at 6 and 9 h, 16.1 at 12 h, and 30.0 at 18 h (Figure 6B). In *Δmg2046*, enzyme activity values were also ∼3.0 at 6 and 9 h, but did not reach any higher than 7.0 at subsequent sampling times. It appears that enzyme activity of WT and *Δmg2046* was similar from 6 to 9 h, but dissolved oxygen level in culture medium subsequently decreased, and nitrate reductase activity in WT strongly increased. Enzyme activity of WT, relative to *Δmg2046*, was threefold higher at 12 and 15 h, and almost 10-fold higher at 18 h.

Enzyme activities of the *Δmg2046* mutant strain were slightly higher at 12 and 15 h than at 6 or 9 h, perhaps because of regulation of MgFnr (Mg2946); however, energy produced by dissimilatory denitrification pathway was limited because cells were entering the late stage of growth. The observed patterns of enzyme activity in the two strains were consistent with those of gene transcription levels. Inhibition of magnetosome synthesis in *Δmg2046* resulted from not only iron deficiency, but also lack of normal energy supply. It is reasonable to conclude that Mg2046 strongly activates transcription of dissimilatory denitrification pathway genes, and that this pathway is essential for magnetosome maturation.

In our previous study, MSR-1 cells showed comparable growth under aerobic and microaerobic conditions, but synthesized magnetosomes only under microaerobic conditions (Wang et al., 2016). High vs. low oxygen supply results in striking differences of MSR-1 metabolism. Understanding the mechanism of biomineralization in MTB requires clarifying the links between cell physiological status and magnetosome formation process. Specific regulators control metabolic pathways of bacteria cultured under differing oxygen concentrations. Two of the best-studied regulators are oxygen- and redox-sensitive proteins Fnr and OxyR. Homologs (or similar proteins) of Fnr and OxyR have been reported in MSR-1. Deletion of MgFnr (Fnr homolog in MSR-1) slightly affected magnetosome formation, mainly by inhibiting nitrous oxide reductase activity under microaerobic conditions (Li et al., 2014b). Deletion of OxyR-like resulted in abnormally shaped magnetosomes, but apparently not through disruption of carbon metabolism (Zhang et al., 2017). Mg2046, the focus of the present study, is clearly involved in basic metabolism and magnetosome formation in MSR-1. In its role as a regulatory protein, its transcription is significantly inhibited by hyperoxia, whereas its function is activated under appropriate microaerobic conditions. Mg2046 function is induced by hypoxic signaling, resulting in activated transcription of dissimilatory denitrification pathway genes, sufficient energy for cell growth and high iron absorption under microaerobic conditions, and consequent magnetosome synthesis.

## Conclusion

This study was focused on the role of novel protein Mg2046 in MSR-1 cell growth and magnetosome synthesis. Major conclusions are as follow. (1) Mg2046 positively regulates magnetosome synthesis in MSR-1, in indirect manner. Absence of Mg2046 disrupts oxygen and iron metabolism, resulting in a series of associated negative effects: reduced iron absorption ability, reduced transcription levels of dissimilatory denitrification pathway genes, inadequate energy supply, and inability to synthesize normal magnetosomes. (2) Mg2046 is a hypoxia-dependent redox regulator involved in maintenance of proper redox state required for magnetosome formation. It appears to exert its regulatory function during early-stage cell growth, and may cooperate with Mg2946 (MgFnr) in regulation of dissimilatory denitrifying pathway, but the two proteins are activated by different oxygen levels. These findings help explain how MSR-1 cells initiate dissimilatory denitrification pathway and overcome energy deficiency under microaerobic conditions, and have broader implications regarding bacterial survival and energy metabolism strategies under hypoxia. Interestingly, Mg2046 is unique to the genus *Magnetospirillum* according to the protein sequence, while the Mg2946 (MgFnr) is widely distributed in almost all bacteria. Mg2046 may thus specially involve in magnetosome formation and dissimilatory denitrification pathway in magnetotactic spiral bacteria.

Our ongoing studies will extend these findings by examining the relationship between Mg2046 and Mg2946, and their roles in regulation of dissimilatory denitrification pathway.

## Author Contributions

YL, XW, and JT conceived the project and designed the experiments. XW, QW, HZ, and YW conducted the experiments and data analysis. JL, JT, YW, and JS provided useful discussion. XW and YL wrote the manuscript. All authors read and approved the finalized manuscript.

## Conflict of Interest Statement

The authors declare that the research was conducted in the absence of any commercial or financial relationships that could be construed as a potential conflict of interest.

## Footnotes

1 www.genoscope.cns.fr/agc/microscope

## Supplementary Material

FIGURE S1 Relative transcription level of *mg2046* gene in *Δmg2046* and complementary strain (*Com2046*), with level in WT as reference (defined as 1). Level in *Com2046* was ∼60-fold higher than in WT.

TABLE S1 qPCR primers used in this study. F: forward primers. R: reverse primers.

TABLE S2 Average magnetosome number and particle size for WT and *Δmg2046*.

TABLE S3 Relative transcription levels of *napF, nirT, norC*, and *nosZ* genes in WT vs. *Δmg2046*.
