# Supplementary material for: Novel Protein Mg2046 Regulates Magnetosome Synthesis in Magnetospirillum gryphiswaldense MSR-1 by Modulating a Proper Redox Status
Source: Front Microbiol. 2019 Jun 26;10:1478. doi: 10.3389/fmicb.2019.01478 (PMC6607277; doi:10.3389/fmicb.2019.01478)
Supplement: TABLE S1 — qPCR primers used in this study. F: forward primers. R: reverse primers. [file Table_1.pdf]

Table S1. qPCR primers used in this study. F: forward primers. R: reverse primers.

**Gene name**		**Primer sequences**
**mam/mms**	**mamY**	F: GAGGAGCCCGCATCGTAT<br>R: AGAGCAATCGGAAGTGAAATGG
**mam/mms**	**mamA**	F: GCCTATCCGTGGCGAAGAA<br>R: TCGGCATCGTAAACCTGCT
**mam/mms**	**mamB**	F: AGGTCGTGTGGTGGGCAT<br>R: CGCTCATCCGCAGGCTTA
**mam/mms**	**mamH**	F: GCAGTCAATGCCAATGTGC<br>R: CCAACCCAGGGACTTAGCG
**mam/mms**	**mamF**	F: GCTATCTGGGCATCCTCTGC<br>R: CGACCACGGACAGCATCA
**mam/mms**	**mms6**	F: GGTTGGCGTTGGGAAGGT<br>R: CATCGCTCTGTGCCGCTT
**mam/mms**	**mamP**	F: TTGTCATTGGGCGGCAG<br>R: ATTGGGCAAGGGCGACT
**mam/mms**	**mamO**	F: TTCCGTGGTAGCGACGATAT<br>R: CAGGGTCGTAATGACATAGCCA
**mam/mms**	**mamT**	F: CGCTGGAGCATCTCATTGA<br>R: ATAGGGCGTCTTGAACTGTGAT
**mam/mms**	**mamS**	F: TCGGCAATGATGTGGGC<br>R: CCAGAACGGACTTGGCGTA
**mam/mms**	**mamE**	F: CGCAATAGCGTGGTTAGCG<br>R: TGAAGCCGTCATTGCGG
**mam/mms**	**mamX**	F: AATGCCAATCCGTGGGAA<br>R: TTCCCTCAACGATGGGCAG
**mam/mms**	**mmsF**	F: TCGGGACGACGAGTTTGTC<br>R: GGAACACCACGGAGACCAA
**iron utilization and storage genes**	**feoB1**	F: GAGGTTCGCCTTTGCCAA<br>R: ATCCAGTCGGTGGGAATGG
**iron utilization and storage genes**	**tolQ**	F: GCTTTACGACCGCATCGG<br>R: GCTCCAGCGTGTCCATCTC
**iron utilization and storage genes**	**tolR**	F: GGTGGATTTGCCCAAGACC<br>R: GGATGAAGATGCGGGTATCG
**iron utilization and storage genes**	**tonB**	F: CGTATCGTCGTGGCAAAGC<br>R: CGGACGAGGTGTTGATTGC
**iron utilization and storage genes**	**exbD**	F: GTCTTCGTCACCATCCGC<br>R: CCATCACCTCCATAAGCCG
**iron utilization and storage genes**	**bfr1**	F: AAGCCCGAAAGTGATAAGCG<br>R: GGCACGCCTTCCAAGAAC
**iron utilization and storage genes**	**brf2**	F: ATGAAAGCCAACCGCACC<br>R: TTCCAGCAGCAAGATGCG

**terminal oxidase genes**	**ctaG**	F: GCCTATCATCGTCCGTTTCG<br>R: TGGTCCGACAGATTGGTGG
	**ctaC**	F: TTGGCGACATTCGTGGC<br>R: TTGCGGTTGGCGTTGTAG
	**ccoQ**	F: CCGACCACCAGCGATTACA<br>R: TGGCGTAGTTCAGCAGGGT
	**ccoN**	F: ATGCTCCACCTGGGCAA<br>R: CGCTTGGGCACGAAATAGT
	**cydB**	F: ATCTGGTGGCTGTTCCTCG<br>R: ACCTGATTGCCGTCCCA
	**cydA**	F: TGTTCGGCATCAACTTCGC<br>R: TTGCTCATACGGTCCCAGC
**dissimilatory denitrification pathway genes**	**napF**	F: TGATGTCGCACAGCCTTAG<br>R: TGATGTCGCACAGCCTTAG
	**nirT**	F: CCATTCACTACACCAACCGTTC<br>R: ATGGCAGTTGCGGCATTC
	**norC**	F: CGGTGTTCGTTGCCTTGA<br>R: CAGACATTGCCCAGTTCCG
	**nosZ**	F: TCGCCACGGTGTCCTTT<br>R: ATCACCTGACCGCTTTGGC
