# Supplementary material for: Novel Protein Mg2046 Regulates Magnetosome Synthesis in Magnetospirillum gryphiswaldense MSR-1 by Modulating a Proper Redox Status
Source: Front Microbiol. 2019 Jun 26;10:1478. doi: 10.3389/fmicb.2019.01478 (PMC6607277; doi:10.3389/fmicb.2019.01478)
Supplement: TABLE S2 — Average magnetosome number and particle size for WT and Δmg2046. [file Table_2.pdf]

Table S2 Average magnetosome number and particle size for WT and Δmg2046.

	**Average magnetosome number**	**Particle size (nm)**
**WT**	18.11±4.89	36.10±9.17
mgΔ2046	8.30±3.02	16.50±5.26
