# Supplementary material for: Novel Protein Mg2046 Regulates Magnetosome Synthesis in Magnetospirillum gryphiswaldense MSR-1 by Modulating a Proper Redox Status
Source: Front Microbiol. 2019 Jun 26;10:1478. doi: 10.3389/fmicb.2019.01478 (PMC6607277; doi:10.3389/fmicb.2019.01478)
Supplement: TABLE S3 — Relative transcription levels of napF, nirT, norC, and nosZ genes in WT vs. Δmg2046. [file Table_3.pdf]

Table S3 Relative transcription levels of *napF*, *nirT*, *norC*, and *nosZ* genes in WT vs. *Δmg2046*.

**Strain**	**sampling time**	***napF***	***nirT***	***norC***	***nosZ***
**WT**	**8 h**	1.03±0.31	1.07±0.43	1.00±0.06	1.00±0.09
	**16 h**	30.98±0.21	5987.79±292.43	1057.73±14.66	310.98±11.74
Δmg2046	**8 h**	0.80±0.2	1.00±0.09	0.96±0.05	0.82±0.13
	**16 h**	8.80±0.25	6.82±0.25	15.07±2.47	6.73±0.52

Note: Level for WT 8 h was used -8h gene transcription level is taken as reference the standard (defined as relative transcription level is 1).
